# Book Notices

**Published:** 1892-02

**Authors:** 


### Book Notices.

REGIONAL ANATOMY IN ITS RELATION TO MEDICINE AND SURGERY: By Geo. McClellan, M. D., Lecturer on Descriptive and Regional Anatomy at the Penn. School of Anatomy; Professor of Anatomy at the Penn. Academy of the Fine Arts, Member of the Association of American Anatomists, etc., in 2 vols.; Vol. I., J. B. Lippincott Co., Philadelphia, 1891.

To write a successful book on Anatomy, in presence of the excellent works of Quain and Gray on Systematic Anatomy, Ellis, Holden and Heath on Regional Anatomy and as dissector's guides, Braune on Sectional Anatomy, and Treeves, Holden and McLachlan on Surgical and Medical Anatomy would be a task of the utmost difficulty; but there is yet room for an Anatomical Atlas which shall be at once complete, well executed, up to date, and sufficiently inexpensive to be within the reach of the average medical student.

Unfortunately, though encouraging the hope that it might do so, McClellan's Regional Anatomy does not fill this gap, nor any other, so far as I can see.

The work is excellently got up, the paper and type unsurpassed, the matter exceedingly pleasant reading, and the plates beautifully artistic productions; but as a student's text-book it is neither sufficiently systematic in its mode of handling the subject nor complete enough in its details. It is essentially superficial in the information it imparts, and even the broad view of anatomy which it presents is described in such a rambling, chaotic and conversational manner, that no man who is not thoroughly familiar with human anatomy could possibly follow the author intelligently. For the purposes of the busy practitioner who may wish to look up some anatomical point of medical or surgical interest the work is equally imperfect and confusing.

The feature of the book is its plates, and these are beautiful enough as colored photographs (though if the coloring be from nature, the advanced state of decay of many of the specimens says little for the author's application of antiseptic practice to the dissecting room) but as plates which are intended to teach anatomy, I have never seen anything more disappointing. Photographs of anything anatomical or pathological are always more or less failures; to produce an anatomical picture which is to teach anything we must have the discrimination of the anatomist combined with the delineating power of the artist. With a dissection before us we pick out and bring into view important points and relations, otherwise little can be made out; and since in a plate handling is impossible, it becomes necessary to impart a slightly diagramatic emphasis to details, else little but the general disposition of the parts will be discernible.

In Dr. McClellan's plates all is delightfully involved in mystery, nothing is definite; not a single dissection is there but gives one the impression that it is only half finished, and from them nothing but vague general ideas can be gleaned.

In short there is no region that is not systematically, thoroughly, and intelligibly described in Ellis' Demonstrations, no medical or surgical points that one does not find better in Gray; and one can learn more anatomy from a glance at a diagram in the latter work than from the most careful study of Dr. McClellan's elaborate plates. Diagrams are very misleading to those who have never dissected; and no plate, however excellent, can do anything but supplement and revive the knowledge gained by the scalpel. W. KEILLER.

---

KEMP & CO.'S PRESCRIBER'S PHARMACOPŒIA:—A synopsis of the more recent remedies, official and unofficial, with a therapeutic index, and a resume of the B. P. additions of 1890. By a member of the Pharmacentical Society of Great Brittain. Revised Reprint, second addition. Kemp & Co., Limited. Wholesale and Manufacturing Chemists. *Bombay*. London office, 84 Leadenhall St., E. C., 1891.

The honest and conscientious pharmacist can be of great service to the physician, and many find their true interest in that direction instead of antagonizing him as some do, by prescribing across the counter instead of sending applicant to the doctor. They can be of service and use, and are, often, by acquainting themselves thoroughly with the properties of drugs, especially new drugs, and preparing them in form for convenient administration. The doctor has not time to investigate the claims of every new drug, nor to make himself acquainted with their physical properties, even. Messrs. Kemp & Co. have aimed to benefit and help the physician in his choice of new drugs, by doing this work for him, they have embodied the result of their research, and investigation of the numerous new remedies in a compact little work under the above head. It is a really valuable work. There are no *proprietary* medicines in it. Many valuable additions have been made to the physician's list of remedies recently. Mr. Kemp calls attention to the fact, that for a long time the new introduction into medicine consisted almost entirely of antipyretics and hypnotics; but in 1890 a wider range was introduced, and embraced some really valuable articles. There is *much* in this book that cannot even be alluded to in a short notice like this.

---

FROM the publishing house of F. A. Davis, Philadelphia, the JOURNAL acknowledges the receipt of the following new books:

Dr. N. S. Davis on "Comsumption."

Coltman on "The Chinese; Medical, Political and Social."

Buret, "On Syphilis in Ancient and Prehistoric Times."

These are all small volumes, very neatly gotten up (price not stated), and all three are on subjects full of interest to the medical man.

Dr. Davis' long experience and observation as a clinician, and his fame as a writer and lecturer, should, and no doubt will, make anything he has to say on consumption not only interesting, but authoritative. As these works are inexpensive, and are really valuable, the JOURUAL would advise its readers, especially the younger men, to send for them and read them.

---

THE PHYSICIAN'S HAND-BOOK for 1891, thirty-fifth year. Albert D. Elmer, M. D. Published by the Putnam Sons, New York.

This splendid pocket companion, having been familiar to the profession so long, an enumeration of its contents would be superfluous. It contains 324 pages, 200 of which are blank, ruled for record of practice, and the balance devoted to a summary of such information as is most needed, especially in emergencies.

This is the nearest approach to a perfect pocket companion for a doctor it is possible to make. Address G. P. Putnam's Sons, N. Y. Price *about* $1.50—not stated.

